# Absolute configuration of fibaruretin B

**DOI:** 10.1107/S1600536811014887

**Published:** 2011-04-29

**Authors:** Hoong-Kun Fun, Abdul Wahab Salae, Ibrahim Abdul Razak, Melati Khairuddean, Suchada Chantrapromma

**Affiliations:** a X-ray Crystallography Unit, School of Physics, Universiti Sains Malaysia, 11800 USM, Penang, Malaysia; b School of Chemical Sciences, Universiti Sains Malaysia, 11800 USM, Penang, Malaysia; c Crystal Materials Research Unit, Department of Chemistry, Faculty of Science, Prince of Songkla University, Hat-Yai, Songkhla 90112, Thailand

## Abstract

The title furan­oditerpenoid, known as fibaruretin B (systematic name: 2β,3α-dihy­droxy-2,3,7,8α-tetra­hydro­penianthic acid lactone), C_20_H_24_O_7_, was isolated from the roots of *Arcangelisia flava*. The absolute configurations at positions 2, 3, 4, 4a, 7, 9, 10a and 10b of fibaruretin B are *S*, *R*, *S*, *R*, *S*, *S*, *S* and *S*, respectively. In the crystal structure, the mol­ecules are linked into infinite chains along the *c* axis by O—H⋯O hydrogen bonds and weak C—H⋯O inter­actions.

## Related literature

For ring conformations, see: Cremer & Pople (1975 ▶). For bond-length data, see: Allen *et al.* (1987 ▶). For background to and activities of furan­oditerpenoids, see: Ito & Furukawa (1969 ▶); Keawpradub *et al.* (2005 ▶); Moody *et al.* (2006 ▶); Nguyen-Pouplin *et al.* (2007 ▶); Su *et al.* (2008 ▶). For a related structure, see: Bakhari *et al.* (1998 ▶). For the stability of the temperature controller used in the data collection, see: Cosier & Glazer (1986 ▶).
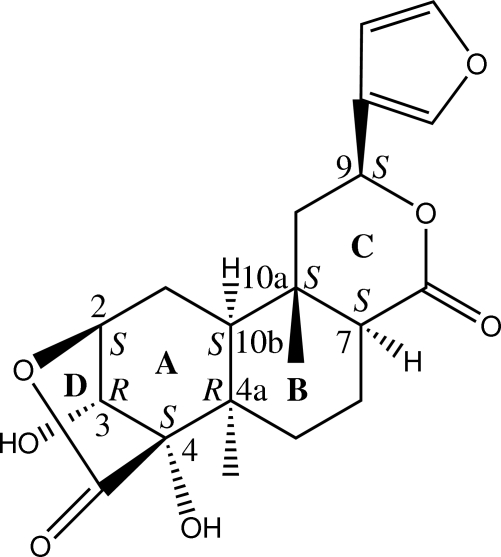


## Experimental

### 

#### Crystal data

C_20_H_24_O_7_
*M* *_r_* = 376.39
Monoclinic, 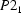

*a* = 7.0942 (2) Å
*b* = 11.7149 (4) Å
*c* = 10.1921 (3) Å
β = 90.805 (1)°
*V* = 846.96 (5) Å^3^
*Z* = 2
Cu *K*α radiation
μ = 0.93 mm^−1^
*T* = 100 K
0.43 × 0.12 × 0.08 mm

#### Data collection

Bruker APEX DUO CCD area-detector diffractometer
Absorption correction: multi-scan (*SADABS*; Bruker, 2009 ▶) *T*_min_ = 0.692, *T*_max_ = 0.926
11247 measured reflections
2645 independent reflections
2641 reflections with *I* > 2σ(*I*)
*R*_int_ = 0.018

#### Refinement

*R*[*F* ^2^ > 2σ(*F* ^2^)] = 0.025
*wR*(*F* ^2^) = 0.066
*S* = 1.09
2645 reflections
254 parameters
1 restraint
H atoms treated by a mixture of independent and constrained refinement
Δρ_max_ = 0.19 e Å^−3^
Δρ_min_ = −0.15 e Å^−3^
Absolute structure: Flack (1983 ▶), with 1098 Friedel pairs
Flack parameter: 0.03 (12)

### 

Data collection: *APEX2* (Bruker, 2009 ▶); cell refinement: *SAINT* (Bruker, 2009 ▶); data reduction: *SAINT*; program(s) used to solve structure: *SHELXTL* (Sheldrick, 2008 ▶); program(s) used to refine structure: *SHELXTL*; molecular graphics: *SHELXTL*; software used to prepare material for publication: *SHELXTL* and *PLATON* (Spek, 2009 ▶).

## Supplementary Material

Crystal structure: contains datablocks global, I. DOI: 10.1107/S1600536811014887/is2703sup1.cif

Structure factors: contains datablocks I. DOI: 10.1107/S1600536811014887/is2703Isup2.hkl

Additional supplementary materials:  crystallographic information; 3D view; checkCIF report

## Figures and Tables

**Table 1:** Hydrogen-bond geometry (Å, °)

*D*—H⋯*A*	*D*—H	H⋯*A*	*D*⋯*A*	*D*—H⋯*A*
O3—H1O3⋯O5^i^	0.86 (3)	2.54 (3)	3.1305 (15)	127 (2)
O4—H1O4⋯O6^i^	0.87 (3)	2.12 (3)	2.9708 (14)	165 (2)
C3—H3*A*⋯O5^i^	0.98	2.39	3.1295 (16)	131
C6—H6*A*⋯O2	0.97	2.43	3.1718 (19)	133
C8—H8*A*⋯O2^ii^	0.98	2.29	3.2113 (19)	157
C19—H19*C*⋯O3	0.96	2.31	2.9489 (18)	124
C20—H20*B*⋯O7^iii^	0.96	2.53	3.4613 (18)	164

Symmetry codes: (i) 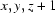; (ii) 
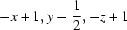
; (iii) 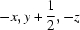.

**Fig. 1.**
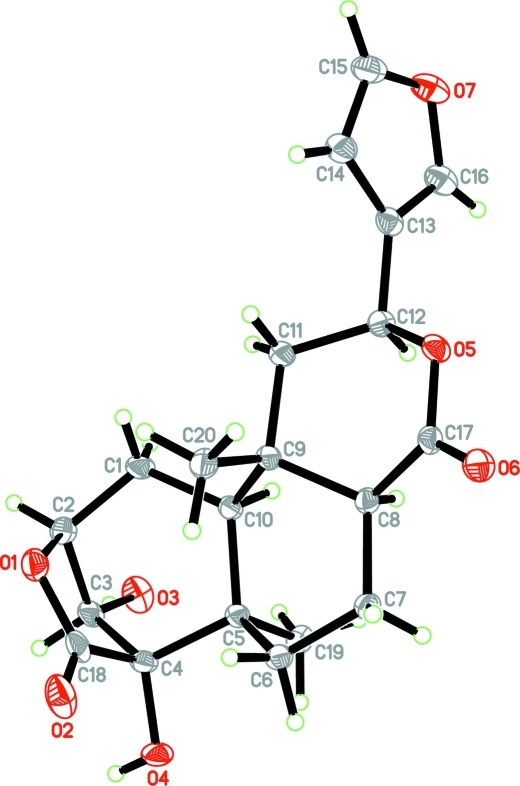
The structure of the title compound, showing 50% probability displacement ellipsoids and the atom-numbering scheme.

**Fig. 2.**
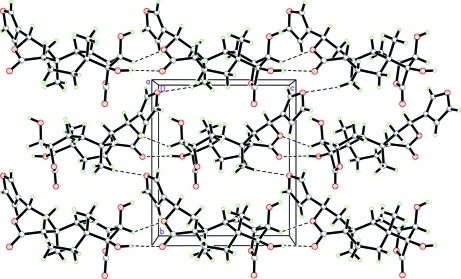
The crystal packing of (I) viewed along the a axis, showing chains along the c axis. Hydrogen bonds are drawn as dashed lines.

## supplementary crystallographic information

### Comment

Furanoditerpenoids are secondary metabolites which were found in Menispermaceae plants such as *Fibraurea chloroleuta* Miers (Ito & Furukawa, 1969), *Fibraurea tinctoria* Lour. (Su *et al.*, 2008) and *Sphenocentrum jollyanum* Pierre (Moody *et al.*, 2006). They were found to possess biological properties such as anti-inflammatory and antimalarial activities. *Arcangelisia flava* (Menispermaceae), commonly called `Khaminkhruea' in the southern Thailand, is widely distributed from Hainan (China), Indo-China, southern peninsular Thailand, peninsular Malaysia, Sumatra, Java, Borneo, the Philipines, Sulawesi, the Northern Moluccas to New Guinea. It is an available medicinal plant used for the treatment of malaria, dysentery and as a tonic. The antimalarial, cytotoxic and antioxidant effects of this plant have been reported (Nguyen-Pouplin *et al.*, 2007; Keawpradub *et al.*, 2005). During the course of our study of bioactive compounds from medicinal plants, the title furanoditerpenoid compound, (I), which is known as fibaruretin B, was isolated for the first time from the roots of *Arcangelisia flava* (Menispermaceae) which were collected from Songkhla province in the southern part of Thailand. The absolute configuration of (I) was determined by making use of the anomalous scattering of Cu Kα radiation with the Flack parameter being refined to 0.03 (12).

The molecule of (I) has four fused rings consisting of one five- and three six-membered rings (*A*/*B*/*C*/*D*) (Fig. 1). The two cyclohexane rings *A* and *B* are *cis* fused, whereas the cyclohexane ring *B* and pyran ring *C* are *trans* fused. The cyclohexane ring *A* (C1–C5/C10) is in an envelope conformation, with the pucker atom C3 (0.4436 (16) Å) and puckering parameter Q = 0.6349 (16) Å, θ = 46.82 (14)° and φ = 115.0 (2)° (Cremer & Pople, 1975). The cyclohexane ring *B* (C5–C10) adopts a twisted boat conformation with puckering parameter Q = 0.7098 (15) Å, θ = 99.25 (12)° and φ = 45.65 (13)°. The pyran ring *C* (O5/C17/C8/C9/C11/C12) is in a boat conformation with puckering parameter Q = 0.7026 (15) Å, θ = 89.67 (12)° and φ = 241.68 (12)°. The tetrahydrofuran ring *D* (O1/C2–C4/C18) is in an envelope conformation, with the pucker atom C3 [-0.2883 (16) Å] and puckering parameter Q = 0.4584 (16) Å and φ = 258.04 (19)°. The furan ring (C13–C16/O7) is planar with an r.m.s. 0.0028 (2) Å and is equatorially attached to the pyran ring *C* with torsion angles O5—C12—C13—C14 = -76.91 (15)° and O5—C12—C13—C16 = 105.01 (16)°. The bond distances in (I) are within normal ranges (Allen *et al.*, 1987) and comparable with the related structure (Bakhari *et al.*, 1998).

The absolute configuration at atoms C2, C3, C4, C5, C8, C12, C9, C10 or positions 2, 3, 4, 4a, 7, 9, 10a and 10b of the fibaruretin B are *S*, *R*, *S*, *R*, *S*, *S*, *S* and *S*, respectively which agree with the previous stereochemistry of fibaruretin B (Su *et al.*, 2008).

In the crystal packing of (I) (Fig. 2), the molecules are linked into chains along the *c* axis through O—H···O hydrogen bonds and C—H···O weak interactions (Fig. 2 and Table 1) and the crystal is stabilized by these interactions.

### Experimental

The air-dried roots of *Arcangelisia flava* (Menispermaceae) (1.8 kg) were extracted with CH_2_Cl_2_ (2 × 10 l) under room temperature. The combined extracts were concentrated under reduced pressure to give a yellow extract (30.1 g) which was subjected to quick column chromatography over silica gel using solvents of increasing polarity from n-hexane to EtOAc to afford 10 fractions (F1–F10). Fraction F9 was further purified by column chromatography using CH_2_Cl_2_–EtOAc (6:4), yielding the title compound as a white solid (275.3 mg). Colorless needle-shaped single crystals of the title compound suitable for X-ray structure determination were recrystallized from CH_2_Cl_2_ by the slow evaporation of the solvent at room temperature after several days (m.p. 539–540 K).

### Refinement

Hydroxy H atom was located from the difference map and refined isotropically. The remaining H atoms were placed in calculated positions with C—H = 0.93 for aromatic, 0.96 Å for CH_3_, 0.97 for CH_2_ and 0.98 Å for CH. The *U*_iso_(H) values were constrained to be 1.5*U*_eq_ of the carrier atom for methyl H atoms and 1.2*U*_eq_ for the remaining H atoms. A rotating group model was used for the methyl groups. The highest residual electron density peak is located at 0.78 Å from H6B and the deepest hole is located at 1.51 Å from C10. 1098 Friedel pairs were used to determine the absolute configuration. Outlier reflection (11 0) was omitted.

### Crystal data

**Table:**

C_20_H_24_O_7_	*F*(000) = 400
*M**_r_* = 376.39	*D*_x_ = 1.476 Mg m^−^^3^
Monoclinic, *P*2_1_	Melting point = 539–540 K
Hall symbol: P 2yb	Cu *K*α radiation, λ = 1.54178 Å
*a* = 7.0942 (2) Å	Cell parameters from 2645 reflections
*b* = 11.7149 (4) Å	θ = 4.3–66.5°
*c* = 10.1921 (3) Å	µ = 0.93 mm^−^^1^
β = 90.805 (1)°	*T* = 100 K
*V* = 846.96 (5) Å^3^	Needle, colourless
*Z* = 2	0.43 × 0.12 × 0.08 mm

### Data collection

**Table:**

Bruker APEX DUO CCD area-detector diffractometer	2645 independent reflections
Radiation source: sealed tube	2641 reflections with *I* > 2σ(*I*)
graphite	*R*_int_ = 0.018
φ and ω scans	θ_max_ = 66.5°, θ_min_ = 4.3°
Absorption correction: multi-scan (*SADABS*; Bruker, 2009)	*h* = −8→8
*T*_min_ = 0.692, *T*_max_ = 0.926	*k* = −11→13
11247 measured reflections	*l* = −11→12

### Refinement

**Table:**

Refinement on *F*^2^	Secondary atom site location: difference Fourier map
Least-squares matrix: full	Hydrogen site location: inferred from neighbouring sites
*R*[*F*^2^ > 2σ(*F*^2^)] = 0.025	H atoms treated by a mixture of independent and constrained refinement
*wR*(*F*^2^) = 0.066	*w* = 1/[σ^2^(*F*_o_^2^) + (0.0393*P*)^2^ + 0.1838*P*] where *P* = (*F*_o_^2^ + 2*F*_c_^2^)/3
*S* = 1.09	(Δ/σ)_max_ = 0.001
2645 reflections	Δρ_max_ = 0.19 e Å^−^^3^
254 parameters	Δρ_min_ = −0.15 e Å^−^^3^
1 restraint	Absolute structure: Flack (1983), with 1098 Friedel pairs
Primary atom site location: structure-invariant direct methods	Flack parameter: 0.03 (12)

### Special details

**Table:**

Experimental. The crystal was placed in the cold stream of an Oxford Cryosystems Cobra open-flow nitrogen cryostat (Cosier & Glazer, 1986) operating at 100.0 (1) K.
Geometry. All esds (except the esd in the dihedral angle between two l.s. planes) are estimated using the full covariance matrix. The cell esds are taken into account individually in the estimation of esds in distances, angles and torsion angles; correlations between esds in cell parameters are only used when they are defined by crystal symmetry. An approximate (isotropic) treatment of cell esds is used for estimating esds involving l.s. planes.
Refinement. Refinement of F^2^ against ALL reflections. The weighted R-factor wR and goodness of fit S are based on F^2^, conventional R-factors R are based on F, with F set to zero for negative F^2^. The threshold expression of F^2^ > 2sigma(F^2^) is used only for calculating R-factors(gt) etc. and is not relevant to the choice of reflections for refinement. R-factors based on F^2^ are statistically about twice as large as those based on F, and R- factors based on ALL data will be even larger.

### Fractional atomic coordinates and isotropic or equivalent isotropic displacement parameters (Å^2^)

**Table:**

	*x*	*y*	*z*	*U*_iso_*/*U*_eq_	
O1	0.09153 (14)	1.02466 (10)	0.68755 (10)	0.0211 (2)	
O2	0.32424 (17)	1.15137 (10)	0.70124 (12)	0.0296 (3)	
O3	0.27902 (16)	0.75672 (11)	0.80552 (11)	0.0252 (3)	
H1O3	0.255 (4)	0.732 (2)	0.883 (3)	0.061 (8)*	
O4	0.55436 (14)	0.96856 (12)	0.80516 (10)	0.0239 (3)	
H1O4	0.527 (3)	0.965 (2)	0.888 (3)	0.046 (6)*	
O5	0.31561 (13)	0.83615 (10)	0.09804 (9)	0.0178 (2)	
O6	0.53283 (13)	0.96975 (11)	0.09604 (9)	0.0206 (2)	
O7	−0.09239 (17)	0.58024 (10)	−0.04095 (11)	0.0275 (3)	
C1	0.08225 (19)	0.83863 (15)	0.58193 (13)	0.0188 (3)	
H1A	−0.0153	0.8680	0.5235	0.023*	
H1B	0.0542	0.7589	0.5981	0.023*	
C2	0.07216 (19)	0.90202 (14)	0.70997 (14)	0.0185 (3)	
H2A	−0.0459	0.8851	0.7547	0.022*	
C3	0.2412 (2)	0.87467 (14)	0.79792 (13)	0.0181 (3)	
H3A	0.2207	0.9052	0.8861	0.022*	
C4	0.39121 (19)	0.94530 (14)	0.72888 (14)	0.0170 (3)	
C5	0.45100 (19)	0.89413 (13)	0.59309 (13)	0.0147 (3)	
C6	0.56241 (19)	0.98757 (13)	0.51939 (14)	0.0162 (3)	
H6A	0.4927	1.0586	0.5247	0.019*	
H6B	0.6821	0.9989	0.5647	0.019*	
C7	0.60174 (18)	0.96286 (14)	0.37404 (14)	0.0163 (3)	
H7A	0.6105	1.0347	0.3272	0.020*	
H7B	0.7225	0.9246	0.3676	0.020*	
C8	0.44977 (18)	0.88869 (13)	0.30780 (13)	0.0145 (3)	
H8A	0.4901	0.8096	0.3218	0.017*	
C9	0.25169 (18)	0.89794 (14)	0.37078 (13)	0.0151 (3)	
C10	0.27476 (18)	0.84594 (14)	0.51044 (13)	0.0147 (3)	
H10A	0.3078	0.7660	0.4940	0.018*	
C11	0.1130 (2)	0.82256 (15)	0.28832 (13)	0.0200 (3)	
H11A	0.0532	0.7684	0.3465	0.024*	
H11B	0.0149	0.8712	0.2516	0.024*	
C12	0.20406 (19)	0.75715 (14)	0.17735 (13)	0.0158 (3)	
H12A	0.2855	0.6969	0.2132	0.019*	
C13	0.0605 (2)	0.70654 (14)	0.08547 (14)	0.0169 (3)	
C14	−0.1096 (2)	0.75809 (15)	0.03551 (14)	0.0214 (3)	
H14A	−0.1519	0.8318	0.0517	0.026*	
C15	−0.1952 (2)	0.67891 (16)	−0.03912 (14)	0.0245 (4)	
H15A	−0.3091	0.6896	−0.0837	0.029*	
C16	0.0642 (2)	0.60018 (15)	0.03548 (15)	0.0226 (3)	
H16A	0.1600	0.5475	0.0508	0.027*	
C17	0.43951 (19)	0.90427 (14)	0.16102 (14)	0.0161 (3)	
C18	0.2742 (2)	1.05332 (15)	0.70502 (14)	0.0196 (3)	
C19	0.5852 (2)	0.79280 (14)	0.61724 (14)	0.0185 (3)	
H19A	0.6860	0.8160	0.6751	0.028*	
H19B	0.6363	0.7681	0.5353	0.028*	
H19C	0.5170	0.7311	0.6564	0.028*	
C20	0.17620 (19)	1.02044 (14)	0.36919 (14)	0.0185 (3)	
H20A	0.0509	1.0215	0.4036	0.028*	
H20B	0.1736	1.0485	0.2807	0.028*	
H20C	0.2567	1.0681	0.4223	0.028*	

### Atomic displacement parameters (Å^2^)

**Table:**

	*U*^11^	*U*^22^	*U*^33^	*U*^12^	*U*^13^	*U*^23^
O1	0.0209 (5)	0.0249 (7)	0.0177 (5)	0.0024 (4)	0.0029 (4)	−0.0014 (5)
O2	0.0385 (6)	0.0192 (7)	0.0313 (6)	−0.0040 (5)	0.0147 (5)	−0.0054 (5)
O3	0.0353 (6)	0.0215 (6)	0.0189 (6)	−0.0015 (5)	0.0068 (5)	0.0065 (5)
O4	0.0183 (5)	0.0407 (7)	0.0125 (5)	−0.0077 (5)	−0.0016 (4)	−0.0034 (5)
O5	0.0203 (5)	0.0222 (6)	0.0109 (4)	−0.0035 (4)	0.0008 (4)	−0.0001 (4)
O6	0.0215 (5)	0.0262 (6)	0.0143 (5)	−0.0042 (5)	0.0021 (4)	0.0028 (4)
O7	0.0346 (6)	0.0253 (7)	0.0225 (6)	−0.0079 (5)	−0.0056 (4)	−0.0038 (5)
C1	0.0175 (7)	0.0248 (9)	0.0141 (6)	−0.0075 (6)	0.0010 (5)	−0.0010 (6)
C2	0.0169 (7)	0.0244 (9)	0.0145 (7)	−0.0046 (6)	0.0041 (5)	0.0001 (6)
C3	0.0209 (7)	0.0223 (9)	0.0112 (7)	−0.0035 (6)	0.0025 (5)	−0.0010 (6)
C4	0.0162 (6)	0.0217 (9)	0.0131 (6)	−0.0044 (6)	−0.0011 (5)	−0.0020 (6)
C5	0.0147 (6)	0.0171 (8)	0.0124 (6)	−0.0016 (6)	0.0005 (5)	−0.0007 (6)
C6	0.0148 (6)	0.0187 (8)	0.0152 (7)	−0.0021 (6)	−0.0003 (5)	−0.0005 (6)
C7	0.0140 (6)	0.0205 (8)	0.0144 (7)	−0.0022 (6)	0.0016 (5)	0.0005 (6)
C8	0.0143 (6)	0.0155 (8)	0.0138 (7)	0.0006 (5)	0.0007 (5)	0.0001 (6)
C9	0.0142 (6)	0.0189 (8)	0.0122 (6)	−0.0022 (6)	0.0012 (5)	−0.0010 (6)
C10	0.0153 (6)	0.0158 (8)	0.0130 (7)	−0.0029 (5)	0.0010 (5)	−0.0015 (5)
C11	0.0159 (6)	0.0296 (9)	0.0144 (7)	−0.0044 (6)	0.0011 (5)	−0.0045 (6)
C12	0.0170 (6)	0.0171 (8)	0.0132 (6)	−0.0010 (6)	0.0001 (5)	0.0029 (6)
C13	0.0197 (7)	0.0185 (8)	0.0124 (6)	−0.0032 (6)	0.0022 (5)	0.0012 (6)
C14	0.0226 (7)	0.0249 (9)	0.0167 (7)	−0.0005 (6)	−0.0008 (5)	0.0000 (6)
C15	0.0248 (7)	0.0310 (10)	0.0177 (7)	−0.0055 (7)	−0.0041 (6)	0.0004 (6)
C16	0.0280 (8)	0.0209 (9)	0.0190 (7)	−0.0036 (6)	−0.0008 (6)	−0.0012 (6)
C17	0.0146 (6)	0.0178 (8)	0.0158 (7)	0.0040 (6)	0.0010 (5)	−0.0013 (6)
C18	0.0254 (7)	0.0210 (9)	0.0125 (6)	−0.0025 (6)	0.0070 (5)	−0.0040 (6)
C19	0.0166 (6)	0.0218 (9)	0.0172 (6)	0.0012 (6)	0.0005 (5)	0.0035 (6)
C20	0.0171 (6)	0.0232 (9)	0.0152 (7)	0.0032 (6)	−0.0005 (5)	0.0016 (6)

### Geometric parameters (Å, °)

**Table:**

O1—C18	1.3482 (19)	C7—C8	1.5338 (19)
O1—C2	1.462 (2)	C7—H7A	0.9700
O2—C18	1.203 (2)	C7—H7B	0.9700
O3—C3	1.409 (2)	C8—C17	1.5078 (19)
O3—H1O3	0.86 (3)	C8—C9	1.5568 (17)
O4—C4	1.4117 (16)	C8—H8A	0.9800
O4—H1O4	0.87 (3)	C9—C20	1.532 (2)
O5—C17	1.3437 (18)	C9—C10	1.5548 (19)
O5—C12	1.4677 (17)	C9—C11	1.5593 (19)
O6—C17	1.2156 (19)	C10—H10A	0.9800
O7—C15	1.367 (2)	C11—C12	1.518 (2)
O7—C16	1.3677 (18)	C11—H11A	0.9700
C1—C2	1.504 (2)	C11—H11B	0.9700
C1—C10	1.5593 (18)	C12—C13	1.4962 (19)
C1—H1A	0.9700	C12—H12A	0.9800
C1—H1B	0.9700	C13—C16	1.347 (2)
C2—C3	1.521 (2)	C13—C14	1.436 (2)
C2—H2A	0.9800	C14—C15	1.340 (2)
C3—C4	1.528 (2)	C14—H14A	0.9300
C3—H3A	0.9800	C15—H15A	0.9300
C4—C18	1.531 (2)	C16—H16A	0.9300
C4—C5	1.5720 (18)	C19—H19A	0.9600
C5—C19	1.539 (2)	C19—H19B	0.9600
C5—C6	1.550 (2)	C19—H19C	0.9600
C5—C10	1.6006 (18)	C20—H20A	0.9600
C6—C7	1.5388 (19)	C20—H20B	0.9600
C6—H6A	0.9700	C20—H20C	0.9600
C6—H6B	0.9700		
			
C18—O1—C2	108.45 (12)	C20—C9—C8	112.23 (12)
C3—O3—H1O3	109.8 (19)	C10—C9—C8	105.44 (11)
C4—O4—H1O4	109.2 (15)	C20—C9—C11	107.87 (11)
C17—O5—C12	117.74 (10)	C10—C9—C11	109.21 (12)
C15—O7—C16	106.07 (12)	C8—C9—C11	107.75 (11)
C2—C1—C10	115.53 (12)	C9—C10—C1	111.57 (11)
C2—C1—H1A	108.4	C9—C10—C5	114.52 (11)
C10—C1—H1A	108.4	C1—C10—C5	117.14 (11)
C2—C1—H1B	108.4	C9—C10—H10A	103.9
C10—C1—H1B	108.4	C1—C10—H10A	103.9
H1A—C1—H1B	107.5	C5—C10—H10A	103.9
O1—C2—C1	110.12 (12)	C12—C11—C9	114.55 (11)
O1—C2—C3	102.95 (12)	C12—C11—H11A	108.6
C1—C2—C3	111.13 (12)	C9—C11—H11A	108.6
O1—C2—H2A	110.8	C12—C11—H11B	108.6
C1—C2—H2A	110.8	C9—C11—H11B	108.6
C3—C2—H2A	110.8	H11A—C11—H11B	107.6
O3—C3—C2	112.76 (13)	O5—C12—C13	105.82 (10)
O3—C3—C4	115.02 (13)	O5—C12—C11	109.30 (12)
C2—C3—C4	99.38 (11)	C13—C12—C11	111.92 (11)
O3—C3—H3A	109.7	O5—C12—H12A	109.9
C2—C3—H3A	109.7	C13—C12—H12A	109.9
C4—C3—H3A	109.7	C11—C12—H12A	109.9
O4—C4—C3	114.97 (12)	C16—C13—C14	105.98 (13)
O4—C4—C18	111.49 (13)	C16—C13—C12	125.95 (15)
C3—C4—C18	98.12 (11)	C14—C13—C12	128.05 (15)
O4—C4—C5	109.22 (11)	C15—C14—C13	106.37 (16)
C3—C4—C5	113.41 (12)	C15—C14—H14A	126.8
C18—C4—C5	109.13 (12)	C13—C14—H14A	126.8
C19—C5—C6	107.67 (11)	C14—C15—O7	110.84 (14)
C19—C5—C4	109.10 (11)	C14—C15—H15A	124.6
C6—C5—C4	107.64 (12)	O7—C15—H15A	124.6
C19—C5—C10	106.79 (12)	C13—C16—O7	110.72 (14)
C6—C5—C10	113.19 (11)	C13—C16—H16A	124.6
C4—C5—C10	112.30 (10)	O7—C16—H16A	124.6
C7—C6—C5	115.73 (12)	O6—C17—O5	118.17 (13)
C7—C6—H6A	108.3	O6—C17—C8	126.71 (13)
C5—C6—H6A	108.3	O5—C17—C8	115.10 (12)
C7—C6—H6B	108.3	O2—C18—O1	121.13 (15)
C5—C6—H6B	108.3	O2—C18—C4	129.41 (14)
H6A—C6—H6B	107.4	O1—C18—C4	109.46 (13)
C8—C7—C6	113.24 (11)	C5—C19—H19A	109.5
C8—C7—H7A	108.9	C5—C19—H19B	109.5
C6—C7—H7A	108.9	H19A—C19—H19B	109.5
C8—C7—H7B	108.9	C5—C19—H19C	109.5
C6—C7—H7B	108.9	H19A—C19—H19C	109.5
H7A—C7—H7B	107.7	H19B—C19—H19C	109.5
C17—C8—C7	113.08 (12)	C9—C20—H20A	109.5
C17—C8—C9	111.68 (11)	C9—C20—H20B	109.5
C7—C8—C9	114.33 (11)	H20A—C20—H20B	109.5
C17—C8—H8A	105.6	C9—C20—H20C	109.5
C7—C8—H8A	105.6	H20A—C20—H20C	109.5
C9—C8—H8A	105.6	H20B—C20—H20C	109.5
C20—C9—C10	114.15 (11)		
			
C18—O1—C2—C1	−94.59 (13)	C2—C1—C10—C9	−121.36 (14)
C18—O1—C2—C3	23.98 (14)	C2—C1—C10—C5	13.4 (2)
C10—C1—C2—O1	64.85 (17)	C19—C5—C10—C9	−116.27 (13)
C10—C1—C2—C3	−48.58 (18)	C6—C5—C10—C9	2.04 (17)
O1—C2—C3—O3	−164.81 (11)	C4—C5—C10—C9	124.18 (13)
C1—C2—C3—O3	−46.95 (16)	C19—C5—C10—C1	110.25 (14)
O1—C2—C3—C4	−42.52 (13)	C6—C5—C10—C1	−131.43 (13)
C1—C2—C3—C4	75.34 (15)	C4—C5—C10—C1	−9.29 (18)
O3—C3—C4—O4	−77.80 (16)	C20—C9—C11—C12	124.47 (13)
C2—C3—C4—O4	161.55 (13)	C10—C9—C11—C12	−110.95 (14)
O3—C3—C4—C18	163.86 (12)	C8—C9—C11—C12	3.10 (18)
C2—C3—C4—C18	43.21 (13)	C17—O5—C12—C13	171.56 (12)
O3—C3—C4—C5	48.87 (17)	C17—O5—C12—C11	50.87 (15)
C2—C3—C4—C5	−71.78 (15)	C9—C11—C12—O5	−51.14 (16)
O4—C4—C5—C19	52.16 (16)	C9—C11—C12—C13	−168.04 (13)
C3—C4—C5—C19	−77.48 (15)	O5—C12—C13—C16	105.01 (16)
C18—C4—C5—C19	174.28 (12)	C11—C12—C13—C16	−136.03 (16)
O4—C4—C5—C6	−64.40 (15)	O5—C12—C13—C14	−76.91 (17)
C3—C4—C5—C6	165.96 (12)	C11—C12—C13—C14	42.1 (2)
C18—C4—C5—C6	57.71 (14)	C16—C13—C14—C15	0.54 (17)
O4—C4—C5—C10	170.36 (13)	C12—C13—C14—C15	−177.84 (14)
C3—C4—C5—C10	40.72 (17)	C13—C14—C15—O7	−0.09 (18)
C18—C4—C5—C10	−67.53 (15)	C16—O7—C15—C14	−0.40 (17)
C19—C5—C6—C7	73.55 (14)	C14—C13—C16—O7	−0.81 (17)
C4—C5—C6—C7	−168.95 (12)	C12—C13—C16—O7	177.62 (13)
C10—C5—C6—C7	−44.25 (16)	C15—O7—C16—C13	0.76 (16)
C5—C6—C7—C8	29.86 (17)	C12—O5—C17—O6	179.82 (13)
C6—C7—C8—C17	155.77 (13)	C12—O5—C17—C8	0.97 (18)
C6—C7—C8—C9	26.47 (18)	C7—C8—C17—O6	−2.4 (2)
C17—C8—C9—C20	−71.31 (15)	C9—C8—C17—O6	128.28 (16)
C7—C8—C9—C20	58.69 (15)	C7—C8—C17—O5	176.37 (12)
C17—C8—C9—C10	163.84 (13)	C9—C8—C17—O5	−52.99 (17)
C7—C8—C9—C10	−66.16 (16)	C2—O1—C18—O2	−174.62 (13)
C17—C8—C9—C11	47.30 (17)	C2—O1—C18—C4	5.10 (14)
C7—C8—C9—C11	177.30 (12)	O4—C4—C18—O2	27.2 (2)
C20—C9—C10—C1	61.69 (15)	C3—C4—C18—O2	148.19 (15)
C8—C9—C10—C1	−174.68 (12)	C5—C4—C18—O2	−93.50 (18)
C11—C9—C10—C1	−59.14 (16)	O4—C4—C18—O1	−152.46 (11)
C20—C9—C10—C5	−74.33 (14)	C3—C4—C18—O1	−31.50 (13)
C8—C9—C10—C5	49.30 (16)	C5—C4—C18—O1	86.81 (13)
C11—C9—C10—C5	164.85 (12)		

### Hydrogen-bond geometry (Å, °)

**Table:**

*D*—H···*A*	*D*—H	H···*A*	*D*···*A*	*D*—H···*A*
O3—H1O3···O5^i^	0.86 (3)	2.54 (3)	3.1305 (15)	127 (2)
O4—H1O4···O6^i^	0.87 (3)	2.12 (3)	2.9708 (14)	165 (2)
C3—H3A···O5^i^	0.98	2.39	3.1295 (16)	131
C6—H6A···O2	0.97	2.43	3.1718 (19)	133
C8—H8A···O2^ii^	0.98	2.29	3.2113 (19)	157
C19—H19C···O3	0.96	2.31	2.9489 (18)	124
C20—H20B···O7^iii^	0.96	2.53	3.4613 (18)	164

Symmetry codes: (i) *x*, *y*, *z*+1;  (ii) −*x*+1, *y*−1/2, −*z*+1; (iii) −*x*, *y*+1/2, −*z*.
